# Cytomorphological patterns and clinical features of presumptive tubercular lymphadenitis patients and their comparison with bacteriological detection methods: a cross-sectional study

**DOI:** 10.1186/s12879-024-09587-4

**Published:** 2024-07-09

**Authors:** Abay Atnafu, Liya Wassie, Melaku Tilahun, Selfu Girma, Mekdelawit Alemayehu, Abenezer Dereje, Gebeyehu Assefa, Tigist Desta, Haymanot Agize, Emnet Fisseha, Yordanos Mengistu, Kassu Desta, Kidist Bobosha

**Affiliations:** 1 Armauer Hansen Research Institute, Addis Ababa, Ethiopia; 2 Department of Medical Laboratory Sciences, College of Health Sciences, Addis Ababa University, Addis Ababa, Ethiopia

**Keywords:** Cytomorphological features, Clinical presentation, GeneXpert, TB lymphadenitis

## Abstract

**Introduction:**

Tuberculous lymphadenitis (TBLN) is an infection of the lymph node caused by *Mycobacterium tuberculosis*. Histological diagnoses of presumptive patients are often accompanied by cytomorphological features. However, the sensitivities of these features are often precluded by the variable degrees of narrative similarities compared to other diagnostic modalities.

**Objective:**

The aim of this study was to investigate and compare the cytomorphological and clinical features of presumptive TBLN patients with bacteriological detection methods.

**Methods:**

A similar cohort of TBLN patients from our previous study who were enrolled prospectively from the ALERT Specialized Hospital, Addis Ababa, Ethiopia, was considered for this analysis. SPSS version 26 was used for data analysis. Descriptive analysis was conducted to characterize the study population using the independent variable and presented with frequency tables. The chi-square test was used to measure the association. A P-value of < 0.05 was considered statistically significant.

**Results:**

Using FNAC, 60/126 (47.6%) of the participants were reported to have features consistent with TB. Of the total FNAC-positive cases, many (30/60 and 27/60) showed pattern B (caseous necrosis only) and pattern C (epithelioid granuloma with caseous necrosis), respectively. Strong concordance was observed in Pattern A (abundant caseous necrosis with few epithelioid macrophages) followed by patterns B and C with GeneXpert and MGIT culture (P value < 0.001). Night sweats and alcohol intake were shown to correlate with positive cases as reported by FNAC (P value = 0.008 respectively), GeneXpert (P value = 0.02 & 0.001), and culture methods (P-value = < 0.001 & 0.002).

**Conclusion:**

Cytomorphological features, particularly patterns A, B, and C, could be considered in the diagnosis of TBLN given their comparable outcomes with bacteriological detection methods. On another note, we recommend that due care and attention be given when treating TBLN patients based solely on clinical presentation, as these diagnostics may be prone to false results, leading to inappropriate administration of anti-TB drugs and other consequences.

**Supplementary Information:**

The online version contains supplementary material available at 10.1186/s12879-024-09587-4.

## Introduction

Infection with *Mycobacterium tuberculosis* (MTB) can also affect organs other than the lung, resulting in a condition known as extrapulmonary tuberculosis (EPTB). The lymph node has been shown by several reports to be the primary organ that appears to be affected, leading to a condition known as tubercular lymphadenitis (TBLN) [1–3]. In Ethiopia, the proportion of TBLN cases can be as high as 73% [4, 5].

In many resource-constrained countries, the availability of sensitive diagnostic tools is often challenging, and this is even more difficult when diagnosing TBLN. In Ethiopia, fine needle aspirate cytology (FNAC) has been used as a primary diagnostic tool for the diagnosis of TBLN [6, 7]. Despite the method being proven simple, inexpensive, and sensitive, several reports have shown the non-specific nature of the method [8]. The basics of FNAC are dependent on finding suggestive features such as granulomatous reactions, features that are often present following a few bacterial or fungal lymphadenitis infections other than TB [9, 10]. Other diagnostic modalities, such as culture and WHO-recommended rapid molecular diagnostics (mWRDS), are often less or not practiced in the routine diagnosis of TBLN despite the provision of bacterial confirmation [11–13].

In the majority of cases, clinicians often rely on interpreting the reported cytomorphological patterns, such as epithelioid granuloma and caseous necrosis, for diagnosing given patients with TBLN [14]. In other instances (at times when FNAC is lacking), clinical presentation, particularly the appearance of a single lymph node swelling, is often considered for classifying patients as having TBLN and providing empirical treatments [15]. Other symptoms, such as fever, tiredness, weight loss, night sweats, cough, and loss of appetite, were also accompanied during TBLN infections [16]. In this study, we compared cytomorphological patterns and clinical presentations against microbiological (culture) and rapid molecular diagnostic techniques (GeneXpert).

## Methods and materials

### Study area and setting

A similar cohort of TBLN patients enrolled prospectively (17) from the ALERT Specialized Hospital in Addis Ababa, Ethiopia, was considered for this analysis. The previous study was a cross-sectional study conducted from March to September 2022, involving 126 patients suspected of having tuberculous lymphadenitis (TBLN) who had previously undergone treatment. The study aimed to investigate the clinical and diagnostic characteristics of this patient population to enhance understanding and improve management strategies for TBLN. The hospital provides FNAC services for five to eight patients with enlarged lymph nodes on a weekly basis. For the study, FNA sample collection adhered to specific criteria to ensure sample integrity and patient safety. Eligible participants included patients of all ages, suspected of having TBLN based on clinical evaluation, and previously treated for tuberculosis, who were willing to provide informed consent. Tuberculous lymphadenitis (TBLN) is characterized by persistent swelling of lymph nodes, typically in the cervical, axillary, or inguinal regions, lasting more than two weeks and often accompanied by systemic symptoms such as fever, night sweats, weight loss, and fatigue.

The FNA procedure was performed by trained healthcare professionals using a sterile 22–25 gauge needle attached to a 10–20 mL syringe, targeting the most prominent and accessible lymph node, preferably from the cervical, axillary, or inguinal regions. The patient’s skin over the lymph node was cleaned with an antiseptic solution before aspiration. The aspirated material was immediately prepared on slides for cytological examination, with a portion placed in a sterile container with the appropriate transport medium for microbiological and molecular tests. All samples were labeled with the patient’s unique identification number, date, and site of aspiration. Post-procedure care included applying a sterile dressing and advising the patient to monitor for any adverse effects. Quality control measures ensured that the samples collected were adequate for analysis, with reassessment and repetition of the FNA if necessary. This comprehensive approach allowed for accurate data collection and analysis, contributing to a better understanding of TBLN and its management.

### Data collection and analysis

The data was collected from March to September 2022, involving 126 patients who were suspected of having tuberculous lymphadenitis (TBLN) and had undergone previous treatment. Sociodemographic and other relevant clinical data were collected using structured questionnaires as summarized in Supplementary Table 1. The data were later captured on Microsoft Excel and exported to the statistical package for the social sciences (SPSS) version 26 for analysis. Descriptive statistics and chi-square tests were used to explain independent variables and measure associations, respectively. A P-value of < 0.05 was considered statistically significant.

### Ethical considerations

Prior to enrollment in the study, written informed consent or assent was obtained from all participants, including parents or guardians for minors, and from children aged 12 to 18 years. Additionally, ethical approval was secured from the AHRI/ALERT Ethics Review Committee.

### Collection of fine needle aspirate (FNA) and slide preparation for cytology

An FNA sample was collected from enlarged lymph nodes or a lump using a 21G needle by an experienced pathologist. Next, two smears were prepared and stained with Wright stain and air dried for further bright field microscopic examination for the presence of suggestive cytomorphological patterns [17, 18]. Cytomorphological patterns were grouped as follows: abundant caseous necrosis with few epithelioid macrophages (Pattern A), caseous necrosis only (Pattern B), epithelioid granuloma with caseous necrosis (Pattern C), and epithelioid granuloma without necrosis (Pattern D). Any leftover FNA sample was collected with cryovials containing 0.5 ml sterile phosphate buffered saline (PBS) for further microbiological confirmation.

### MGIT 960 culture detection system

Any leftover FNA samples collected using sterile PBS were transported to the AHRI TB laboratory for MGIT 960 TB culture testing. Samples were initially decontaminated using standard decontamination and concentration procedures, and the pellet was resuspended using 1.5 ml sterile PBS for inoculation. Before inoculation, 800 µl of PANTA-supplement mixture was added to the MGIT tube, 500 µl of the resuspended sample was inoculated into the MGIT tube, and the tubes were gently mixed and incubated at 37 °C until growth was detected (Supplementary Fig. 1) using the MGIT 960 culture detection system [19].

### GeneXpert

The remaining FNA aliquot (~ 500 µl) was mixed with 1.5 ml of the diluent and incubated for 15 min at room temperature. Using transfer pipettes, the whole mixture was transferred into the GeneXpert cartridge, which was then loaded into the GeneXpert machine [20]. The result will be displayed after 2 h (Supplementary Fig. 2).

### Quality control

Quality control for the MGIT-960 culture system involved regular calibration of the instrument, using approved media and reagents within their expiration dates, and running both positive and negative control strains with each batch of samples to verify performance. Positive controls included a known Mycobacterium tuberculosis strain, while negative controls used sterile water or a known negative strain. QC procedures also entailed strict aseptic techniques during inoculation, regular monitoring of the instrument for any malfunctions, and thorough documentation of all QC activities, including outcomes and any corrective actions taken. Similarly, QC for the GeneXpert system included calibrating the instrument as per manufacturer guidelines, inspecting cartridges and reagents for defects, and running external positive and negative controls with each test batch. Positive controls contained known concentrations of Mycobacterium tuberculosis DNA, while negative controls used sterile water or buffer. Adherence to strict sample processing protocols and detailed record-keeping of QC tests and corrective actions ensured the reliability and accuracy of results for both systems.

## Results

### Distribution of various cytomorphological patterns in socio-demographic characteristics

The overall proportion of TB lymphadenitis by the FNAC was 60/126 (47.6%), of which only one case had abundant caseous necrosis with few epithelioid macrophages (Pattern A), seen in a child below the age of one year. The highest number of FNAC patterns were caseous necrosis (Pattern B) and epithelioid granuloma with caseous necrosis (Pattern C), mostly seen in the 21–30 age group (13/60 (21.6%)). A larger proportion of females had FNAC patterns consistent with TB, 36/60 (60%) compared to men, 24/60 (40%) (Table 1).

**Table 1:** Distribution of cytomorphological patterns among TBLN patients across age and sex at ALERT Hospital in 2022 (*N* = 126)

		FNAC patterns						Total	P-value	
		Pattern A	Pattern B	Pattern C	Pattern D	Features inconsistent with TB	Inconclusive			
Age (years)	0–10	1	1	0	0	4	0	6	0.356	
	11–20	0	8	6	0	15	0	29		
	21–30	0	13	13	2	22	1	51		
	31–40	0	6	5	0	12	0	23		
	41–50	0	1	1	0	2	0	4		
	51–60	0	0	1	0	4	0	5		
	61–70	0	1	0	0	4	0	5		
	71–80	0	0	0	0	2	0	2		
	81–90	0	0	1	0	0	0	1		
Total		1	30	27	2	65	1	126		
Sex	Female	0	22	13	1	40	1	77	0.798	
	Male	1	8	14	1	25	0	49		
Total		1	30	27	2	65	1	126		

### Characteristics of lymph nodes

The majority of participants (100 out of 126, or 79.4%) presented with a single lymph node, with 74 of these cases (58.7%) occurring on the unilateral left side. The cervical lymph nodes appeared to be the most commonly affected site (99/126, 78.6%). In 60/126 (47.6%) study participants, the swelling duration lasted 1–10 weeks, and the swelling progressed slowly in 59/126 (46.8%) participants. Node tenderness during examination was noted in only 18/126 (14.3%) participants. The size of the lymph node was 1*1 cm in 53/126 (42.1%), and it was mobile in 69/126 (54.8%) participants. Approximately 50% of the lymph nodes were matted (Supplementary Table 2).

### Distribution of clinical features across FNAC and bacteriological detection methods

A larger proportion of the study participants presented with poor appetite and body weakness (72/126 (57.1%) and 75/126 (59.5%), respectively). Out of 126 patients, 61 had night sweats, and 25 had coughs. Among the entire study participants who presented with poor appetite, 34/72 (47.2%), 24/72 (33.3%), and 23/72 (31.9%) were found to be positive using FNAC, GeneXpert, and MGIT, respectively. Approximately 30/75 (40%) of the participants who presented with body weakness were culture positive, and this association was statistically significant (*P* = 0.001); likewise, 40/75 (53.3%) and 30/75 (40%) were shown to be positive using FNAC and GeneXpert, respectively.

Overall, 38/61 (62.3%), 31/61 (50.8%), and 29/61 (47.5%) of the participants with night sweats were detected as positive using FNAC (P value = 0.008), GeneXpert (P value = < 0.001), and MGIT culture (P value = < 0.001), respectively, and the associations were statistically significant (*P* < 0.05). Similarly, participants with a history of alcohol intake had a strong association with a positive microbiologic test result (P-value < 0.05) Supplementary Table 3.

### Comparison of cytomorphological patterns with bacteriological detection methods

One of the participants presenting with Pattern A using FNAC was found to be positive using GeneXpert and MGIT. Among participants with Pattern B, 20/30 (66.6%) and 18/30 (60%) were found to be positive using GeneXpert and culture, respectively (P-value < 0.001). On the other hand, from those presenting with Pattern C, 8/27 (29.6%) and 11/27 (40.7%) were found to be negative using GeneXpert and culture, respectively. Among participants with Pattern D, only a small number tested negative, with 1 out of 2 (50%) negative using GeneXpert and 2 out of 2 (100%) negative using culture (Table 2).

**Table 2:** Comparison of cytomorphological patterns with bacteriological detection methods

		FNAC patterns						Total	p-value
		Pattern A	Pattern B	Pattern C	Pattern D	Features inconsistent with TB	Inconclusive		
GeneXpert	MTB detected - Medium	0	1	2	1	0	0	4	< 0.001
	MTB detected - Low	0	1	3	0	1	0	5	
	MTB detected - Very low	1	18	14	0	0	0	33	
	MTB not detected	0	10	8	1	64	1	84	
Total		1	30	27	2	65	1	126	
MGIT	Positive	1	18	16	0	1	0	36	< 0.001
	Negative	0	12	11	2	64	1	90	
Total		1	30	27	2	65	1	126	

### Distribution of clinical features and various cytomorphological patterns

Among all participants with a previous history of anti-TB treatment (16/126), 12/16 (75%) had night sweats (P-value = 0.02). From a total of 25 participants with cough, 6/25 (24%) had a previous treatment history (*P* = 0.05). On the other hand, weight loss was not observed in the majority of the participants (62.5%) with a previous history of anti-TB treatment. A cytomorphological feature not consistent with TB was also observed in a few cases (3/16) among the previously treated cases (Table 3).

**Table 3:** Distribution of clinical presentation and various cytomorphological patterns in patients with a history of previous anti-TB treatment

		Previous treatment History		Total	X^2^	P value
		Yes	No			
Weight loss	Yes	6	56	62	1	0.99
	No	10	54	64		
	Total	16	110	126		
Night sweating	Yes	12	49	61	5.12	0.02
	No	4	61	65		
	Total	16	110	126		
Poor appetite	Yes	10	62	72	0.21	0.64
	No	6	48	54		
	Total	16	110	126		
Body weakness	Yes	11	64	75	0.65	0.425
	No	5	46	51		
	Total	16	110	126		
Cough	Yes	6	19	25	3.6	0.05
	No	10	91	101		
	Total	16	110	126		
Alcohol intake	Yes	4	4	8	10.7	0.001
	No	12	106	118		
	Total	16	110	126		
FNAC Patterns	Pattern A	1	0	1	17.4	0.01
	Pattern B	6	24	30		
	Pattern C	6	21	27		
	Pattern D	0	2	2		
	Features in consistent with TB	3	62	65		
	Inconclusive	0	1	1		
	Total	16	110	126		

### Distribution of clinical presentation and various cytomorphological patterns in GeneXpert RIF-resistant cases

Among the total patients who had a history of night sweating, 4/61 (6.6%) showed a rifampicin (RIF) drug resistance pattern, and this was statistically significant (P-value = 0.02). Among a few participants who had a history of alcohol intake, 2/8 (25%) showed RIF resistance patterns (*P* = 0.001). A similar number of RIF-resistant cases (2/4 (50%)) also showed caseous necrosis only and epithelioid granuloma with caseous necrosis patterns. No RIF-resistant cases were observed in other patterns suggestive of TB (Table 4).

**Table 4:** Distribution of clinical presentation and various cytomorphological patterns in GeneXpert RIF-resistant cases

		GeneXpert RIF resistance			Total	X^2^	P value
		Detected	Not detected	Indeterminate			
Weight loss	Yes	3	58	1	62	2.2	0.143
	No	1	63	0	64		
	Total	4	121	1	126		
Night sweating	Yes	4	56	1	61	5.5	0.02
	No	0	65	0	65		
	Total	4	121	1	126		
Poor appetite	Yes	2	70	0	72	1.4	0.3
	No	2	51	1	54		
	Total	4	121	1	126		
Body weakness	Yes	3	72	0	75	1.9	0.68
	No	1	49	1	51		
	Total	4	121	1	126		
Cough	Yes	1	23	1	25	4.2	0.1
	No	3	98	0	101		
	Total	4	121	1	126		
Alcohol intake	Yes	2	6	0	8	13.3	0.001
	No	2	115	1	118		
	Total	4	121	1	126		
FNAC Patterns	Pattern A	0	1	0	1	8.8	0.03
	Pattern B	2	28	0	30		
	Pattern C	2	24	1	27		
	Pattern D	0	2	0	2		
	Features in consistent with TB	0	65	0	65		
	Inconclusive	0	1	0	1		
	Total	4	121	1	126		

## Discussion

Lymphadenitis is the most common clinical presentation of EPTB. It may present as unilateral single or multiple painless lumps, mostly located in the neck’s posterior cervical or supraclavicular region [21]. In Ethiopia. Approximately 20–30% of all TB cases are caused by extrapulmonary TB, with 60% of all EPTB cases involving the lymph nodes and the pleural membrane [22]. In this study, we included 126 participants who presented with enlarged lymph nodes, most of whom were from the urban cities of Ethiopia. In our study, we observed women to be slightly more affected by TBLN than men, with a higher proportion of cervical lymph nodes and the presence of a single node. This was also consistent with an earlier observation conducted in India, where more than 81% of the total participants had shown a single lymph node [23]. The typical clinical presentation for TBLN is said to be a matted nontender mass, usually with accompanying symptoms such as low-grade fever, weight loss and loss of appetite [21, 24]. From our study population, we also noted a larger proportion of the lymph nodes being matted.

The diagnosis of tuberculous lymphadenitis (TBLN) employs several microbiological detection methods to ensure accuracy and reliability. Fine needle aspiration (FNA) cytology is often the initial step, where samples are examined for characteristic features such as granulomas and caseous necrosis. Acid-fast bacilli (AFB) staining is performed on these samples to identify Mycobacterium tuberculosis directly. Culturing the aspirated material in liquid media using the Mycobacteria Growth Indicator Tube (MGIT) 960 system enhances the detection of viable mycobacteria, providing a definitive diagnosis through growth and subsequent identification. Molecular methods, particularly the GeneXpert MTB/RIF assay, amplify and detect Mycobacterium tuberculosis DNA, offering rapid and sensitive confirmation of the presence of the bacteria, along with information on rifampicin resistance.

In a resource-poor country such as Ethiopia, where there is limited access to FNAC and other bacteriological confirmation tests, evaluation of TBLN-suspected patients with clinical presentation becomes an available option. However, reliance on clinical symptom screening with little laboratory support for diagnosis may lead to overdiagnosis in endemic areas [25]. In our study, participants presenting with body weakness predominated, followed by those with poor appetite. However, they were shown to have a poor consistency of narrating the positive cases when compared with the bacteriological detection method. This may imply that patients treated with anti-TB drugs based on the above clinical presentation could be at risk of developing adverse consequences. On the other hand, participants presenting with night sweats in our study showed a significant concordance with FNAC and bacteriological detection methods. On a similar note, participants with alcohol intake also appeared to have a significantly increased number of concordances with positive cases reported by the FNAC. However, confirmation with bacteriological detection methods prior to administration of anti-TB drugs has always to be considered, as these clinical features often appear to be lacking in most instances. These observations possibly indicate how relying solely on the clinical evaluation could be misleading, resulting in inappropriate treatment administration in TBLN patients. Other studies have also reported the unreliable feature of clinical diagnosis of TBLN [26]. However, in our study, clinical features such as night sweating and alcohol intake better predicted positivity, as observed in their rate of cytomorphological patterns suggestive of TB.

Tubercular lymphadenitis (TBLN) is diagnosed if epithelioid cell granuloma is present, with or without multinucleate giant cells and necrosis, on FNAC and/or on tissue biopsy [24]. In the absence of granuloma or necrosis, the cytology/tissue findings are misleading [27]. Even in cases with granulomas, confirmative diagnosis, including bacteriologic examination, is essential for the initiation of treatment because of the presence of various granulomatous inflammatory conditions [28].

In our study, the four FNAC features (abundant caseous necrosis with few epithelioid macrophages, caseous necrosis only, epithelioid granuloma with caseous necrosis, and epithelioid granuloma without necrosis) of TBLN were observed in only 60 participants out of the 126, making a 47.6% in the diagnosis of TBLN. A higher proportion of positivity rate, 30/60 (50%) and 27/60 (45%), was predominated by caseous necrosis only and epithelioid granuloma with caseous necrosis in this study. A slightly higher positivity rate in the epithelioid granuloma with caseous necrosis and a lower positive rate of caseous necrosis were reported by only two studies conducted in India [29, 30]. Another study conducted in India has shown a result consistent with this study, a domination of caseous necrotic material only [7]. Based on the above evidence, in a setting where FNAC is the only available diagnostic tool, caseous necrosis only and epithelioid granuloma with caseous necrosis could be considered a dominating feature suggestive of TB.

Lymph nodes are the most typical extrapulmonary presentations of TB. A strong index of suspicion and the use of several different diagnostic modalities are required for reaching accurate diagnosis and differentiation of other differential diagnoses. Although it is not realistic or viable to use all diagnostic techniques, our results have shown that it is better to approach patients with TBLN with molecular diagnostic methods to supplement routine diagnostic tools to improve diagnosis and minimize missed cases of TBLN. In our study, to rule out other causes of granulomatous inflammatory conditions, comprehensive evaluations of microbiological techniques were performed to identify bacterial and fungal causes of swollen lymph node, and specific stains for non-tuberculous mycobacteria (NTM), though not identified, was tried to be ruled out by inoculating the FNA specimen into LJ slant supplemented with pyruvate. Histopathological examination of biopsy samples can differentiate between sarcoidosis, which shows non-caseating granulomas, and TB, characterized by caseating granulomas. Additionally, clinical correlation with patient history, symptoms, and imaging studies further aids in excluding other potential causes of granulomatous inflammation, ensuring an accurate diagnosis of TBLN.

Considering the relatively small sample size in our study, we recognize the necessity for further investigation to collect additional data, which would support a more definitive approach in diagnosing tuberculous lymphadenitis (TBLN). Despite the valuable insights gained, it has become evident that relying solely on a single diagnostic method is insufficient for accurate diagnosis and clinical decision-making. The limitations inherent in using only one diagnostic approach were highlighted by the variability in sensitivity and specificity, potential for false positives or negatives, and the inability to capture the full spectrum of the disease. These findings underscore the importance of incorporating multiple diagnostic modalities, such as combining cytological, microbiological, molecular, and radiological methods, to enhance diagnostic accuracy and provide a more comprehensive assessment of TBLN. Consequently, future studies should adopt a multi-faceted diagnostic strategy and include larger, more diverse patient cohorts to validate these findings and improve the robustness of TBLN diagnosis and management.

## Conclusion

From the findings in this study, we observed that among the four cytomorphological features, Pattern A, Pattern B, and Pattern C were consistent with the other two bacteriological detection methods in diagnosing the positive cases. On the other hand, among the clinical features that were presented by the study participants, night sweating was observed in the majority of the positive cases, with a significant number. Body weakness was observed with a significant number in those positive cases reported by the MGIT culture method. Alcohol intake was also shown with a significant number in positive cases reported by the three methods. Hence, among the cytomorphological and clinical features presented in this study, the abovementioned features appeared to be likely to occur in TBLN patients.

Therefore, cytomorphological features, particularly patterns A, B, and C, could be considered in the diagnosis of TBLN given their comparable outcomes with bacteriological detection methods. On another note, we recommend that due care and attention be given when treating TBLN patients based solely on clinical presentation, as these diagnostics may be prone to false results, leading to inappropriate administration of anti-TB drugs and other consequences.

### Electronic supplementary material

Below is the link to the electronic supplementary material.

Supplementary Material 1

Supplementary Material 2

Supplementary Material 3

Supplementary Material 4

Supplementary Material 5

## Data Availability

The underlying data are available upon request. Point of contact for data request: ayatab.ayele7@gmail.com.

## Abbreviations

AFB: Acid Fast Bacilli
AHRI/ALERT: Armauer Hansen Research Institute/All Africa Leprosy, Tuberculosis and Rehabilitation Training
ALERT: All Africa Leprosy, Tuberculosis and Rehabilitation Training
DNA: Deoxyribonucleic Acid
EPTB: Extra Pulmonary Tuberculosis
FNA: Fine Needle Aspirate
FNAC: Fine Needle Aspirate Cytology
IRB: Institutional Review Board
LJ: Lowenstein Jensen
MGIT: Mycobacterial Growth Indicator Tube
MTB: Mycobacterium Tuberculosis
mWRDS: WHO-recommended rapid molecular diagnostics
NTM: Non Tuberculous Mycobacteria
PANTA: Polymyxin B, Amphotericin B, Nalidixic acid, Trimethoprim, and Azlocillin
PBS: Phosphate Buffered Saline
QC: Quality Control
RIF: Rifampicin
SPSS: Statistical Package for Social Science
