# Supplementary material for: Cytomorphological patterns and clinical features of presumptive tubercular lymphadenitis patients and their comparison with bacteriological detection methods: a cross-sectional study
Source: BMC Infect Dis. 2024 Jul 9;24:684. doi: 10.1186/s12879-024-09587-4 (PMC11234654; doi:10.1186/s12879-024-09587-4)
Supplement: Supplementary file 1 — Supplementary Material 1 [file 12879_2024_9587_MOESM1_ESM.docx]

Supplementary table 1: Socio-demographic characteristics of study participants in ALERT hospital, Addis Ababa, Ethiopia.

| **Variable** | **Frequency** | **Percent (%)** |
| --- | --- | --- |
| **Age in years** | | |
| 0-10 | 6 | 4.8 |
| 11-20 | 29 | 23 |
| 21-30 | 51 | 40.5 |
| 31-40 | 23 | 18.3 |
| 41-50 | 4 | 3.2 |
| 51-60 | 5 | 4 |
| 61-70 | 5 | 4 |
| 71-80 | 2 | 1.6 |
| 81-90 | 1 | 0.8 |
| **Gender** | | |
| Female | 77 | 61.1 |
| Male | 49 | 38.9 |
| **Marital status** | | |
| Married | 61 | 48.4 |
| Single | 59 | 46.8 |
| Living with partner | 6 | 4.8 |
| **Living area** | | |
| Urban | 117 | 92.9 |
| Rural | 9 | 7.1 |
| **Educational status** | | |
| 1-6 | 59 | 46.8 |
| Uneducated | 67 | 53.2 |
| **Occupational status** | | |
| Housewives | 38 | 30.2 |
| Governmental employee | 29 | 23 |
| Student | 29 | 23 |
| Unemployed | 16 | 12.7 |
| Farmer | 9 | 7.1 |
| Children | 4 | 3.2 |
| Private | 1 | 0.8 |
