# Supplementary material for: Cytomorphological patterns and clinical features of presumptive tubercular lymphadenitis patients and their comparison with bacteriological detection methods: a cross-sectional study
Source: BMC Infect Dis. 2024 Jul 9;24:684. doi: 10.1186/s12879-024-09587-4 (PMC11234654; doi:10.1186/s12879-024-09587-4)
Supplement: Supplementary file 2 — Supplementary Material 2 [file 12879_2024_9587_MOESM2_ESM.docx]

Supplementary table 2: Lymph node characteristics of the study participants at ALERT Hospital

| **Variable** | **Frequency** | **Percent (%)** |
| --- | --- | --- |
| **Number of lymph node** | | |
| Single | 100 | 79.4 |
| Multiple | 26 | 20.6 |
| Location of node |  |  |
| Anterior and posterior cervical | 5 | 4 |
| Anterior left sided | 15 | 11.9 |
| Unilateral left sided | 74 | 58.7 |
| Unilateral right sided | 32 | 25.4 |
| **Distribution pattern of lymph node** | | |
| Cervical | 99 | 78.6 |
| Axillary | 16 | 12.7 |
| Inguinal | 4 | 3.2 |
| other | 7 | 5.6 |
| **Lymph node swelling duration** | | |
| 1-10 weeks | 60 | 47.6 |
| 11-20 weeks | 28 | 22.2 |
| >20 weeks | 38 | 30.2 |
| **Rate of increase in swelling** | | |
| Fast | 12 | 9.5 |
| Moderate | 55 | 43.7 |
| Slow | 59 | 46.8 |
| **Lymph node tenderness** | | |
| Tender | 18 | 14.3 |
| Non tender | 108 | 85.7 |
| **Lymph node size** | | |
| 1*1 cm | 53 | 42.1 |
| 2*1 cm | 20 | 15.9 |
| 2*2 cm | 26 | 20.6 |
| 2*3 cm | 10 | 7.9 |
| 3*3 cm | 8 | 6.3 |
| 4*3 cm | 6 | 4.8 |
| 4*4 cm | 3 | 2.4 |
| **Lymph node mobility** | | |
| Mobile | 69 | 54.8 |
| Non mobile | 57 | 45.2 |
| **Condition of lymph node** | | |
| Discrete | 16 | 12.7 |
| Fluctuant | 42 | 33.3 |
| Matted | 63 | 50.0 |
| Soft | 5 | 4.0 |
