# Supplementary material for: Cytomorphological patterns and clinical features of presumptive tubercular lymphadenitis patients and their comparison with bacteriological detection methods: a cross-sectional study
Source: BMC Infect Dis. 2024 Jul 9;24:684. doi: 10.1186/s12879-024-09587-4 (PMC11234654; doi:10.1186/s12879-024-09587-4)
Supplement: Supplementary file 3 — Supplementary Material 3 [file 12879_2024_9587_MOESM3_ESM.docx]

|  | | Cytomorphological patterns | | | | | | p-value | GeneXpert | | p-value | MGIT | | p-value |
| --- | --- | --- | --- | --- | --- | --- | --- | --- | --- | --- | --- | --- | --- | --- |
|  |  | Pattern A | Pattern B | Pattern C | Pattern D | Features not consistent with TB | Inconclusive |  | Negative | Positive |  | Negative | Positive |  |
| Weight loss | Yes | 1 | 18 | 12 | 2 | 28 | 1 | 0.67 | 38 | 24 | 0.39 | 40 | 22 | 0.09 |
|  | No | 0 | 12 | 15 | 0 | 37 | 0 |  | 46 | 18 |  | 50 | 14 |  |
| Total | | 1 | 30 | 27 | 2 | 65 | 1 |  | 84 | 42 |  | 60 | 36 |  |
| Poor appetite | Yes | 1 | 17 | 14 | 2 | 37 | 1 | 0.877 | 48 | 24 | 0.414 | 49 | 23 | 0.333 |
|  | No | 0 | 13 | 13 | 0 | 28 | 0 |  | 36 | 18 |  | 41 | 13 |  |
| Total | | 1 | 30 | 27 | 2 | 65 | 1 |  | 84 | 42 |  | 90 | 36 |  |
| Body weakness | Yes | 1 | 20 | 18 | 1 | 34 | 1 | 0.151 | 45 | 30 | 0.053 | 45 | 30 | 0.001 |
|  | No | 0 | 10 | 9 | 1 | 31 | 0 |  | 39 | 12 |  | 45 | 6 |  |
| Total | | 1 | 30 | 27 | 2 | 65 | 1 |  | 84 | 42 |  | 90 | 36 |  |
| Night sweating | Yes | 1 | 17 | 18 | 2 | 22 | 1 | 0.008 | 30 | 31 | <0.001 | 32 | 29 | <0.001 |
|  | No | 0 | 13 | 9 | 0 | 43 | 0 |  | 54 | 11 |  | 58 | 7 |  |
| Total | | 1 | 30 | 27 | 2 | 65 | 1 |  | 84 | 42 |  | 90 | 36 |  |
| Cough | Yes | 1 | 6 | 7 | 0 | 11 | 0 | 0.611 | 12 | 13 | 0.14 | 14 | 11 | 0.056 |
|  | No | 0 | 24 | 20 | 2 | 54 | 1 |  | 72 | 29 |  | 76 | 25 |  |
| Total | | 1 | 30 | 27 | 2 | 65 | 1 |  | 84 | 42 |  | 90 | 36 |  |
| Alcohol intake | Yes | 0 | 6 | 2 | 0 | 0 | 0 | 0.008 | 2 | 6 | 0.006 | 2 | 6 | 0.002 |
|  | No | 1 | 24 | 25 | 2 | 65 | 1 |  | 82 | 36 |  | 88 | 30 |  |
| Total | | 1 | 30 | 27 | 2 | 65 | 1 |  | 84 | 42 |  | 90 | 36 |  |

Supplementary Table 3: Comparison of the clinical characteristics of the study participants with Cytomorphological patterns, GeneXpert, and Culture method
