# Supplementary figures and images for: Cytomorphological patterns and clinical features of presumptive tubercular lymphadenitis patients and their comparison with bacteriological detection methods: a cross-sectional study
Source: BMC Infect Dis. 2024 Jul 9;24:684. doi: 10.1186/s12879-024-09587-4 (PMC11234654; doi:10.1186/s12879-024-09587-4)

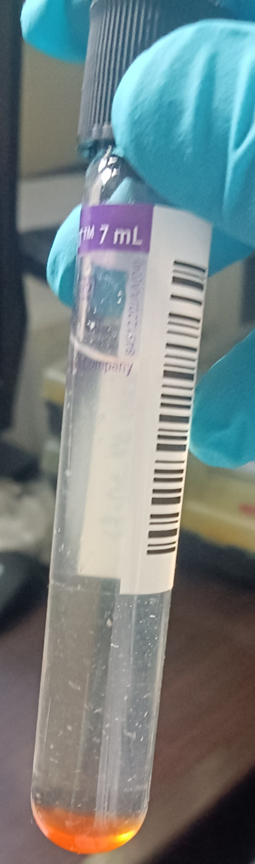

Supplement: Supplementary file 4 — Supplementary Material 4 [file 12879_2024_9587_MOESM4_ESM.tif]

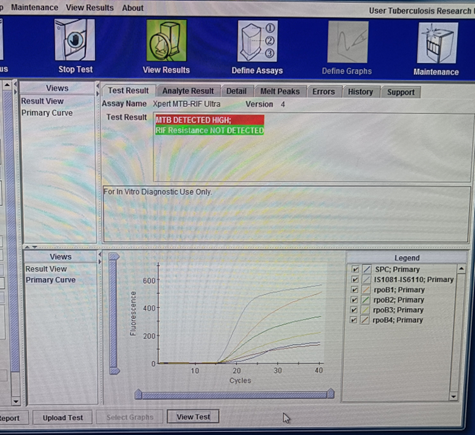

Supplement: Supplementary file 5 — Supplementary Material 5 [file 12879_2024_9587_MOESM5_ESM.tif]
